# Radioactive Tracing of Testosterone Reveals Minimal Formation of 5α-DHT in SGBS Cells and Human Primary Adipocytes

**DOI:** 10.1210/jendso/bvaf087

**Published:** 2025-05-16

**Authors:** Andrea Andress Huacachino, Cátia F Marques, Clementina Mesaros, Trevor M Penning

**Affiliations:** Department of Biochemistry & Biophysics, Perelman School of Medicine, University of Pennsylvania, Philadelphia, PA 19104, USA; Center of Excellence in Environmental Toxicology, Perelman School of Medicine, University of Pennsylvania, Philadelphia, PA 19104, USA; Center of Excellence in Environmental Toxicology, Perelman School of Medicine, University of Pennsylvania, Philadelphia, PA 19104, USA; Department of Systems Pharmacology & Translational Therapeutics, Perelman School of Medicine, University of Pennsylvania, Philadelphia, PA 19104, USA; Center of Excellence in Environmental Toxicology, Perelman School of Medicine, University of Pennsylvania, Philadelphia, PA 19104, USA; Department of Systems Pharmacology & Translational Therapeutics, Perelman School of Medicine, University of Pennsylvania, Philadelphia, PA 19104, USA; Department of Biochemistry & Biophysics, Perelman School of Medicine, University of Pennsylvania, Philadelphia, PA 19104, USA; Center of Excellence in Environmental Toxicology, Perelman School of Medicine, University of Pennsylvania, Philadelphia, PA 19104, USA; Department of Systems Pharmacology & Translational Therapeutics, Perelman School of Medicine, University of Pennsylvania, Philadelphia, PA 19104, USA

**Keywords:** adipocytes, androgen metabolism, hyperandrogenism, PCOS

## Abstract

Hyperandrogenism is associated with polycystic ovary syndrome (PCOS), acne, and alopecia. In PCOS, subcutaneous fat has been shown to contribute to hyperandrogenism through increased testosterone (T) production which is accompanied by an increase in the intra-adipose 5α-dihydrotestosterone (5α-DHT):T ratio. However, whether 5α-DHT is produced in isolated adipocytes is uncertain. Here we investigated the ability of subcutaneous human adipocytes to synthesize and inactivate 5α-DHT in a model of subcutaneous white adipocytes, Simpson-Golabi-Behmel syndrome (SGBS) cells, and primary adipocytes. We quantified the transcripts of genes involved in the biosynthesis of 5α-DHT (*AKR1C3, SRD5A1, SRD5A2*, and *HSD17B6*) and the inactivation of 5α-DHT (*AKR1C1* and *AKR1C2*). We found that genes that inactivate 5α-DHT were more abundantly transcribed than genes that biosynthesize 5α-DHT. This trend was reflected by radioisotope tracing. We developed a radiochromatographic method involving high-performance liquid chromatography and in-line detection of radioactive analytes with precision and accuracy within the 15% tolerance allowable by the US Food and Drug Administration criteria for analytical assays. The lower limit of detection and quantification for 5α-DHT was 3.4 pg and 15 pg, respectively. The formation of 5α-DHT was barely detectable when starting with either 10 nM T or 3α-androstanediol (3α-diol). Conversely, 5α-DHT was rapidly metabolized to 3α-diol but not 3β-diol. 3α-Diol was the major metabolite despite comparable levels of *AKR1C1* and *AKR1C2* transcripts. The same result was observed in both cell lines. Our data reveal that adipocytes do not biosynthesize 5α-DHT from testosterone. By contrast, 5α-DHT is rapidly metabolized by AKR1C2 in subcutaneous adipocytes.

Hyperandrogenism refers to elevated levels of serum androgens and is most associated with polycystic ovary syndrome (PCOS) [1] but can contribute to other pathologies such as acne, hirsutism, and alopecia [2]. Testosterone (T) is mainly produced in the Leydig cells of the testis in men and in lower quantities in the ovaries of women. In men and women, dehydroepiandrosterone (DHEA), DHEA-sulfate, 4-androstene-3,17-dione, and 11β-hydroxy-4-androstene-3,17-dione are adrenal androgens that can be converted to T and 11-Keto-testosterone (11K-T) and their 5α-dihydroderivatives, respectively, in peripheral tissues in an intracrine or paracrine-dependent manner [3-5]. In PCOS the ovaries contribute to the hyperandrogenism observed due to stimulation by luteinizing hormone but peripheral tissues may convert adrenal precursors to T as well. By contrast, 5α-dihydrotestosterone (5α-DHT) is mainly produced in peripheral target tissues [6, 7].

Adipose tissue (AT) also contributes to androgen production [8]. Increased levels of abdominal AT, comprised of both subcutaneous AT (SAT) and visceral AT (VAT), are associated with the prevalence of PCOS [9]. Of the two, VAT has higher androgenic activity, which is thought to contribute to insulin resistance [10]. However, SAT has also been associated with insulin resistance, particularly, from the abdominal depot [11, 12]. Microdialysis experiments showed that DHEA is converted to T and 5α-DHT in intra-SAT from PCOS patients but not controls, suggesting that this process may contribute to the hyperandrogenic profile in PCOS. Subsequent experiments showed that primary subcutaneous adipocytes and preadipocyte Simpson-Golabi-Behmel syndrome (SGBS) cells, a preadipocyte model, increased T production from 4-androstene-3,17-dione (4AD) when stimulated with insulin, and this was mediated by induction of aldo-keto reductase family 1 member C3 (AKR1C3) [13, 14]. The production of T by this route was inhibited by up to 80% using 3 different AKR1C3 inhibitors. SGBS cells also express supraphysiological levels of HSD11B1, which in transfection studies can convert 4AD to T in comparable amounts to transfected AKR1C3. In SGBS cells the HSD11B1 inhibitor AZD4017 was able to block 60% of T formation as well [15]. Clearly, AKR1C3 and HSD11B1 both may play a role in T biosynthesis in SGBS cells. Of note is the ability of HSD11B1 to convert the 11-keto-T (a potent androgen) to 11β-hydroxy-T (a weak androgen) and regulate ligand occupancy of the androgen receptor (AR). However, these prior studies did not test the ability of fully differentiated adipocytes to biosynthesize 5α-DHT.

In adipocytes a number of pathways to 5α-DHT are possible (Fig. 1). In the canonical pathway, DHEA can be converted to 4AD by 3β-hydroxy-Δ-5-steroid dehydrogenase/isomerase (HSD3B1). 4AD is then converted to T by AKR1C3 or HSD11B1, which is subsequently reduced to 5α-DHT, the most potent ligand for the AR, by 5α-reductase type 1/2 (SRD5A1/2). In the Δ^5^-Adiol pathway, DHEA can be converted to 5-androstenediol (Δ^5^-Adiol) by AKR1C3 and then metabolized by HSD3B1 to T en route to 5α-DHT. In the 5α-Adione pathway, 4AD can be converted to 5α-androstane-3,17-dione (5α-Adione) by SRD5A1/2 and then converted to 5α-DHT via AKR1C3, a pathway that bypasses T altogether [16]. In the backdoor pathway, androsterone can be converted to 3α-diol by AKR1C3 and then oxidized to 5α-DHT by HSD17B6 [17-19]. Whether adipocytes produce 5α-DHT by these routes is an important question since 5α-DHT increases inflammatory cytokines in human adipocytes [20, 21].

**Figure 1.**
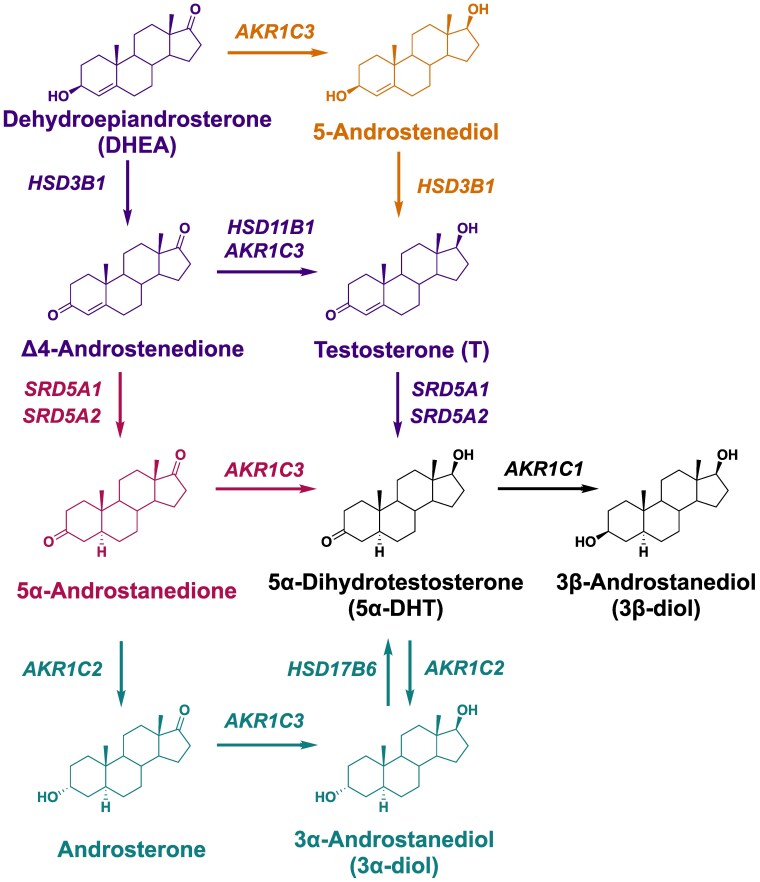
Potential pathways for 5α-DHT biosynthesis and its metabolism in human adipocytes. The canonical pathway is depicted in purple, the 5α-Adione pathway in pink, the backdoor pathway in teal, and the Δ^5^-Adiol pathway in brown. Gene names are given in italics.

Characterization of androgen metabolism at the metabolite level is challenging, and often inference is made based on transcript levels alone. However, this leaves uncertainties regarding enzyme expression and activity. Historically, androgen quantification has been subject to various limitations. One such limitation is the lack of a UV chromophore or fluorophore. 5α-DHT, the most potent androgen, is difficult to detect due to these limitations. Methods to detect 5α-DHT can rely on its derivatization to form hydrazones, which can be detected by UV/Vis [22], but lack sensitivity for cell-based work. Picolinate derivatization can improve ionization and when coupled with liquid chromatography–tandem mass spectrometry (LC-MS/MS) detection [23] is sensitive in the low picogram (pg) range but may not be readily available in all laboratories and increases sample manipulation. Others have reported the use of radiochromatography with high-performance liquid chromatography (HPLC) coupled with in-line β-RAM detection with subsequent validation of radioactive peaks by comigration with authentic standards by thin-layer chromatography. However, this approach has not been used to determine the precision and accuracy of the method [16, 24, 25]. Here we present a method to quantify underivatized 5α-DHT, its metabolites 3α-diol and 3β-diol, and its precursor T using HPLC-β-RAM to provide precise quantification with precision and accuracy in the picogram range. To demonstrate the robustness of the method, we characterized analyte identity using unlabeled reaction products by LC-MS. We used this method to investigate the synthesis and metabolism of 5α-DHT in fully differentiated SGBS cells and differentiated primary human adipocytes.

## Materials and Methods

### Chemicals and Reagents

All reagents were American Chemical Society grade or higher and were commercially available as follows: T (Steraloids, catalog No. S1655371), epitestosterone (Steraloids, catalog No. S1757678), dehydroepiandrosterone (Steraloids, catalog No. 70804), dihydrotestosterone (Steraloids, catalog No. 146109), androsterone (Steraloids, catalog No. A2420-000), epiandrosterone (ICN biomedicals, catalog No. 159923), 5-Androstene-3β,17β-Diol (Steraloids, catalog No. 126466), 3α-Androstanediol (Steraloids, catalog No. 142032), 3β-Androstanediol (Steraloids, catalog No. 127425), [1,2-^3^H]-5α-dihydrotestosterone, 60.0 Ci/mmol (American Radiolabeled Chemicals, CAS: 521-18-6, catalog No. ART0769, lot: 240318), [4-^14^C]-testosterone, 50.0 mCi/mmol (Fisher Chemical, CAS: 633-32-9, catalog No. NEC101050UC, lot: 3590023), ), [2,3,4-^13^C_3_]-dehydroepiandrosterone (Cambridge Isotope Laboratories, catalog No. CLM-10549-0.001), [2,3,4-^13^C_3_]-testosterone (Cambridge Isotope Laboratories, catalog No. 9164-PK), [23,4-^13^C_3_]-dihydrotestosterone (Cambridge Isotope Laboratories, catalog No. CLM-9146-D), paraformaldehyde (Sigma-Aldrich, CAS: 30525-89-4, catalog No. 158127, lot: STBL0660), NADPH tetrasodium salt hydrate (Sigma Aldrich, CAS: 2646–71–1, catalog No. 101078240001, lot: 58124426), NADH (Sigma Aldrich, CAS: 606-68-8, catalog No. 10107735001, lot: 46675921), 4-(dimethylamino)pyridine (Aldrich, CAS: 1122-58-3, catalog No. 107700-5G, lot: MKCJ5395), 2-picolinic acid (Aldrich, CAS: 98-98-6, catalog No. P42800-100G, lot: WXBC1726V), 2-methyl-6-nitrobenzoic anhydride (TCI, CAS: 434935-69-0, catalog No. M1439, lot: PCRCK), and triethylamine (Sigma, CAS: 121-44-8, catalog No. 90340-25ML).

Cell culture reagents were as follows: Dulbecco's phosphate-buffered solution (PBS) (Gibco, catalog No. 14190-136), 0.25% trypsin-EDTA (Gibco, catalog No. 25200-056), penicillin-streptomycin (10 000 U/mL) (Gibco, catalog No. 15140-122), HyClone fetal bovine serum (FBS) (Cytiva, catalog No. SH30071.03, Lot: AH30005075), Dulbecco's Modified Eagle Medium (DMEM) nutrient mixture F-12 (Gibco, catalog No. 11330-032), biotin (Sigma, catalog No. B4639, lot: SLCF3557), D-pantothenic acid hemicalcium salt (Sigma, catalog No. P5155, lot: SLBZ6233), apo-transferrin human (Sigma, catalog No. T2252, lot: SLCK2671), human recombinant insulin (Gibco, CAS: 11061-68-0, catalog No. 12585014), cortisol (Sigma-Aldrich, CAS: 50-23-7, catalog No. H6909), 3,3′,5-triiodo-L-thyronine sodium salt (Sigma, CAS: 55-06-1, catalog No. T6397), dexamethasone (Sigma-Aldrich, CAS: 50-02-2), 3-isobutyl-1-methylxanthine (Sigma-Aldrich, CAS: 28800-58-4), rosiglitazone (Cayman chemical company, catalog No. 71740, lot: 0487968-125), Oil Red O (Sigma, catalog No. 09755-25G, lot: 018k0669), and Hoechst 33342 (Thermo, catalog No. 62249, lot: TJ2649192).

All molecular biology reagents were commercially available as follows: radioimmunoprecipitation assay buffer (Pierce/Thermo, catalog No. 89900), Protease Inhibitor (Sigma, catalog No. P8340), BCA Assay Kit (Pierce/Thermo, catalog No. 23227), 0.45 μm nitrocellulose membranes (Bio-Rad, catalog No. 1620115), Ponsceau S staining (Sigma, catalog No. P7170-1L), blocking buffer (Bio-Rad, catalog No. 1706404), rabbit anti-human AKR1C1/1C2 (Abcam, catalog No. ab179448), murine antihuman AKR1C3 (Sigma, catalog No. AB_476751), murine anti-human α-Tubulin (Novus Biologicals, catalog No. NB100-690SS), rabbit-IgG-HRP (Santa Cruz; catalog No. sc-2357), murine-IgG_K_-HRP (Santa Cruz, catalog No. sc-516102), and ECL Western Blotting Substrate (Thermo, catalog No. 32106).

All solvents were HPLC grade as follows: dimethyl sulfoxide (Sigma Aldrich, CAS: 67-68-5, catalog No. 34869-100ML, lot: SHBJ4596), acetonitrile (Fisher chemical, CAS: 75-05-8, catalog No. A998-4, lot: 211149), diethyl ether (Sigma Aldrich, CAS: 60-29-7, catalog No. 309966-1L, lot: SHBP9911), methanol (Fisher chemical, CAS: 67-56-1, catalog No. A545-4), and formic acid (Fisher chemical, CAS: 64-18-6, catalog No. A117-50).

### Adipocyte Cell Culture and Differentiation

SGBS preadipocytes (a gift from the Wabitsch laboratory at the University of Ulm, Germany) were grown and differentiated into mature adipocytes over 14 days based on the protocol from the Wabitsch laboratory [26, 27]. Preadipocytes were passaged in growth media (DMEM/F12, 10% FBS, 1% penicillin/streptomycin, 33 µM biotin, 17 µM pantothenic acid) and were maintained at a growth rate of 2 generations over 3 days. In preparation for differentiation, preadipocytes were seeded into 6-cm dishes (Corning) and grown for 3 days to reach 500 000 cells at 90% confluency. After 3 days of growth, cells were washed with PBS and quick differentiation was initiated with differentiation medium (growth medium without FBS) containing 10 µg/mL apo-transferrin, 20 nM insulin, 100 nM cortisol, 200 pM 3,3′,5-triiodo-L-thyronine sodium salt, 25 nM dexamethasone, 250 µM 3-isobutyl-1-methylxanthine, and 2 µM rosiglitazone. After 4 days, the quick differentiation medium was replaced with 3FC media (differentiation medium minus 25 nM dexamethasone, 250 µM 3-isobutyl-1-methylxanthine, and 2 µM rosiglitazone).

Primary preadipocytes were obtained by ATCC (ATCC, PCS-210-010, lot: 70004210) from a 17-year-old Hispanic female patient via lipoaspirate. Primary cells were maintained, passaged, and differentiated according to the ATCC protocol [28]. Briefly, cells were cultured with fibroblast basal medium (ATCC, PCS-201-030) containing growth kit-low serum (ATCC, PCS-201-041, lot: 80526241) (5 ng/mL recombinant human FGF-β 7.5 mM L-glutamine, 50 mg/mL ascorbic acid, 1 mg/mL hydrocortisone/hemisuccinate, 5 mg/mL recombinant human insulin, and 2% FBS). When preadipocytes reached 80% confluence, they were split into 6-well plates (Corning) with approximately 5000 cells/cm^2^ with 2 mL of fibroblast basal medium. After 2 days of growth, the adipocyte differentiation procedure was started by adding 2 mL of adipocyte differentiation initiation medium (ATCC, PCS-500-050, lot: 80316241). After 2 days, the initiation media was replaced with adipocyte differentiation maintenance medium (ATCC, PCS-500-050, lot: 80316241). This step was repeated every 3 days until adipocytes reached full maturity by 14 days.

### Red Oil Lipid Quantification Assay

Cells were phenotyped using Oil Red O (Sigma) staining for lipid droplet formation. Cells (and a plate blank) were fixed by washing the cell plate with PBS, followed by placing 2 mL of 4% (w/v) paraformaldehyde (PFA) followed by quick aspiration. Then, an additional 2 mL of PFA was added and left to incubate for 30 minutes. The PFA was aspirated after incubation. To stain the cells, 2 mL of 60% isopropanol was added and incubated for 5 minutes. After aspirating, 2 mL of 6:4 v/v (Oil Red O 3 mg/mL in isopropanol and water) was added and incubated for 15 minutes. After incubation, the plates were washed with deionized water 4 times. Then 2 mL of water was added to each plate. To stain the nucleus, 100 μL of Hoechst stain was added and left to incubate for 10 minutes in minimal light. The cells were imaged using a Biotek Cytation 5 plate reader using color brightfield and nuclei viewed under DAPI (4′,6-diamidino-2-phenylindole) filter cube.

For Oil Red O quantification, the plates were washed with deionized water twice. Then 1 mL of isopropanol was added. Plates were scrapped to ensure dye was fully extracted. Extracted samples were stored in 13 × 100 mm Pyrex glass tubes at −20 °C. In a 96-well plate, 100 μL of the extract was added and absorbance was read at 510 nm. All readings were baseline-subtracted.

### Reverse-Transcription Quantitative Polymerase Chain Reaction Assays

RNA was extracted with 600 µL of lysis buffer with 2-mercaptoethanol using the RNeasy mini kit (Qiagen, catalog No. 74104, lot: 57202853) and treated with DNase (Qiagen, catalog No. 79254, lot: 57201215) as per the manufacturer’s protocol. RNA concentrations were determined with a Nanodrop ND-2000. RNA was reverse-transcribed to complementary DNA (cDNA) using a High-Capacity RNA to cDNA kit (Applied Biosystems/Thermo, catalog No. 4387406, lot: 2754580). Expression was measured by QuantiTect SYBR Green PCR Master Mix (Qiagen, catalog No. 204143, lot: 178032546) in the Chromo4 System. Calibration curves of log fg target gene vs Ct value were linear over 9 orders of magnitude using serial dilutions of the standard, which indicates that the amount of product doubles with each cycle indicating a PCR amplification efficiency equal to 10-^1/slope^ – 1 or 100%.

### *GAPDH* Primers

Primers have been previously reported by our laboratory [29]. The conditions for the real-time polymerase chain reaction (PCR) for glyceraldehyde-3-phosphate dehydrogenase (*GAPDH*) were 95 °C for 15 minutes followed by 40 cycles of 95 °C for 15 seconds (denaturation) and 58 °C for 30 seconds (annealing and extension) Standard curves were generated using full-length standards (2 500 000-0.025 fg) as previously described by our laboratory [29].

### AKR1C1, AKR1C3, and HSD17B6 Primers

*AKR1C1, AKR1C3*, and *HSD17B6* primers have been previously reported by our laboratory [29]. The conditions for the real-time PCR for *AKR1C1*, *AKR1C3*, and *HSD17B6* were 95 °C for 15 minutes followed by 40 cycles of 94 °C for 15 seconds (denaturation), 57 °C for 30 seconds (annealing temperature), and 72 °C for 30 seconds (extension temperature). All standard curves were generated using full-length standards (2 500 000-0.025 fg) as previously described by our laboratory [29].

### *AKR1C2* Primers

*AKR1C2* primers have been previously reported by our laboratory [29]. The conditions for the real-time PCR for *AKR1C2* were 95 °C for 15 minutes followed by 40 cycles of 94 °C for 15 seconds (denaturation), 62 °C for 30 seconds (annealing temperature) and 72 °C for 30 seconds (extension temperature). Standard curves were generated using full-length standards (2 500 000-0.025 fg) as previously described by our laboratory [29].

### SRD5A1, SRD5A2, GLUT4 Primers

*SRD5A1* primers (forward: 5′- CCTAAGGAA TCTCAGAAAACCAGG—3′ reverse: 5′—GCATAGCCACACCACTCCATGA—3′), *SRD5A2* primers (forward: 5′—TCCCAGCACTTGCATTTG—3′ reverse: 5′—TTCCGAGAT TTGGGGTAGTC—3′) and *GLUT4* primers (forward: 5′—CCATCCTGATGACTGTGGCTCT—3′ reverse: 5′—GCCACGATGAACCAAGGAATGG—3′) were purchased from Penn Oligo and designed using Origene and their cDNA sequence in NCBI. The conditions for the real-time PCR for *SRD5A1, SRD5A2*, and *GLUT4*, were 95 °C for 15 minutes followed by 40 cycles of 95 °C for 30 seconds (denaturation), and 60 °C for 30 seconds (annealing and extension temperature). PCR product standards (2 500 000-0.025 fg) were generated for *SRD5A1* with the following: forward primer 5′- TACGGGCATCGGTGCTTAAT-3′ and reverse primer 5′- GTGAAGAAAGCAAAAGCCGC-3′ from HEK293 cells. Standards for *SRD5A2* and *GLUT4* were purchased from Penn Oligo:

*SRD5A2*: 5′—CTCCCAGCACTTGCATTTGCATTTTTCTCACTTTGTTTCCTTGGGCTGCGAGCTTTTCACCACCATAGGTTCTACCTCAAGATGTTTGAGGACTACCCCAAATCTCGGAAA—3′,

*GLUT4*: 5′—GCTCCTGCTGGAGCGAGTTCCAGCCATGAGCTACGTCTCCATTGTGGCCATCTTTGGCTTCGTGGCATTTTTTGAGATTGGCCCTGGCC—3′.

The correction factors for PCR product standards due to difference in molecular weight between full-length and PCR product standards were as follows: SRD5A2 (6.94), SRD5A1 (2.21), and GLUT4 (17.25).

### Androgen Metabolism Assay

Metabolism of T, 5α-DHT, and 3α-diol were conducted in both adipocyte cell lines. Fully differentiated SGBS adipocytes were treated with 10 nM [^14^C]-T (2.7 pCi/pmol), 10 nM [^3^H]-5α-DHT (73.8 pCi/pmol), 10 nM [^3^H]-3α-diol (73.8 pCi/pmol), or control vehicle (0.01% dimethyl sulfoxide) in 6 mL of media containing DMEM/F12, 1% penicillin/streptomycin, 33 µM biotin, and 17 µM pantothenic acid. Starting at 0 hours, 1 mL of media was collected from each 6-cm plate at 24-hour intervals for 96 hours (4 days). Samples were stored at −20 °C until extraction. At the final time point, protein was harvested to normalize data by protein concentration.

Fully differentiated primary adipocytes were treated with 10 nM [^14^C]-T (2.7 pCi/pmol), [^3^H]-5α-DHT (73.8 pCi/pmol), [^3^H]-3α-diol (73.8 pCi/pmol), or control vehicle (0.01% dimethyl sulfoxide) in 3 mL of maintenance media. Starting at 0 hours, 0.5 mL of media was collected from each well at 24-hour intervals for 96 hours (4 days). Samples were stored at −20 °C until extraction. At the final time point, protein was harvested to normalize readings by protein concentration.

### Enzymatic Synthesis of [1,2-^3^H_2_]-3α-diol and [1,2-^3^H_2_]-3β-diol

[1,2-^3^H_2_]-3α-diol was synthesized from [1,2-^3^H_2_] 5α-DHT using recombinant AKR1C9. [1,2-^3^H_2_]-3β-diol was synthesized from [1,2-^3^H_2_] 5α-DHT using recombinant AKR1D1 E120H. The AKR1D1 E120H mutant has a single point mutation to eliminate the 5β-reductase activity of the enzyme and generate an enzyme that has only 3β-HSD activity [23, 30].

For the 3α-diol reactions, the reaction contained 100 mM potassium phosphate buffer (pH 6.0), 4% acetonitrile, 1.6 mM NADH, AKR1C9 (9.3 ng/µL), and 10 μM [^3^H_2_]-5α-DHT (0.03 μCi/pmol) in a total volume of 1 mL. For the 3β-diol reaction, the reaction system was composed of 100 mM potassium phosphate buffer (pH 6.0), 4% ACN, 1 mM NADPH, AKR1D1 E120H mutant (14 ng/mL), and 10 μM [^3^H_2_]-5α-DHT (0.03 μCi/pmol*)*. Both reactions were incubated at 37 °C for 1 hour. After incubation, the solution was extracted with 2 mL of diethyl ether by shaking for 30 minutes, centrifuging for 10 minutes, and extracting the top organic layer. The extraction was repeated twice. The extracts were dried by a Savant SPD121P SpeedVac Concentrator before being reconstituted in 60% acetonitrile. The purity of the enzymatically synthesized radioactive standards was determined by β-RAM-HPLC (Supplementary Fig. S1 [31]).

### Androgen Extraction

Each time point was taken by aliquoting 1 mL (SGBS) or 0.5 mL (primary) of media into a glass vial and storing at −20 °C. Once all samples were collected, androgens were extracted with 2 mL diethyl ether by shaking for 30 minutes, followed by centrifugation for 10 minutes, and removing the top organic layer. The extraction was repeated twice. Extracts were pooled, combined, and dried using a Savant SPD121P SpeedVac Concentrator and the residue was reconstituted in 100 µL of 60% acetonitrile.

### Discontinuous β-RAM-HPLC Detection of Androgens

Radioactive samples were analyzed on a Waters Zorbax ODS 5 um 4.6 × 250 mm analytical C8 column (PN: 880952-706, SN: USL0030722, lot: 805055) on a Waters Alliance 2695 Separation Module using a constant flow rate of 0.5 mL/min with the solvents water (A) and acetonitrile (B). The HPLC method started at 95% A for 4 minutes then increased to 45% B over 4 minutes. The method remained isocratic at 45% B for 47 minutes then increased to 95% B over 1 minute, ran at 95% B for 4 minutes, and then returned to 95% A over 1 minute. The column was reequilibrated at 95% A for 4 minutes.

Compounds were detected using an IN/US Systems β-RAM model 3 Radio-HPLC detector with a scintillation liquid flow rate of 1.5 mL/min (3:1 scint:elutant) and dwell time of 1 second. EcoLite scintillation cocktail (MP Biomedicals, catalog No. 882475, lot: U1124204664) was used as the scintillation cocktail. The β-RAM detector was operated with a wide channel (0-1000 HV) to detect both isotopes simultaneously. Chromatograms were analyzed by Waters Empower Pro software, and results were analyzed in GraphPad Prism 9.0.

### Stable-Isotope Dilution Liquid Chromatography Tandem Mass Spectrometry Identification of Androgens

[^13^C_3_]-3α-diol and [^13^C_3_] 3β-diol standards were synthesized as described earlier (Supplementary Fig. S2 [32]). Samples were derivatized by picolinate acid as described previously by us [23]. Androgens were identified using a stable-isotope dilution (SID)-LC-MS/MS previously developed in the laboratory [23]. Briefly, a Vanquish UPLC system was coupled to a TSQ Atlis Plus MS. Picolinic acid derivatized androgens, epitestosterone (Epi-T), T, DHEA, androsterone (Ad), 5α-DHT, Epiandrosterone (Epi-Ad), 3α-diol, and 3β-diol were chromatographically separated using a C18 column [23]. Solvent A was 0.05% (v/v) formic acid in water, solvent B was 0.05% formic acid in 4:6 acetonitrile:methanol, and the flow rate was 0.25 mL/min. The LC method was slightly modified from previously reported and began at 20% B for 1 minute, then increased to 60% B over 5 minutes and maintained at 60% B for 20 minutes. B then increased to 95% over 15 minutes and was maintained at 95% for 5 minutes. B was then decreased to 20% over 5 minutes. The column was reequilibrated with 20% B for 15 minutes. The MS was operated with the following parameters: ion polarity: positive; electrospray voltage: 4000 V; sheath gas: 28; auxiliary gas: 15; ion transfer capillary temperature: 350 °C; vaporizer temperature: 100 °C; and collision gas: 1.5 mTor (argon). Xcalibur 4.6 software (Thermo Scientific) was used for data acquisition and processing (Supplementary Fig. S3A [33]).

### Scintillation Counting

Detection of radioactivity in aqueous samples was accomplished by liquid scintillation counting. The aqueous layer following androgen extraction described earlier was spun for 10 minutes. Then, a 10-μL aliquot was added to a 6.5-mL plastic SnapTwist scintillation vial (Simport, catalog No. S207) with 4 mL of EcoLite scintillation cocktail. The vial was capped and inverted to mix 10 times. Tri-Carb 2100TR Liquid Scintillation Analyzer (Packard, A Canberra Company) measured counts per minute in each sample, which were converted to disintegrations per minute by machine efficiency calculated by using machine standards for ^3^H and ^14^C. Results were analyzed using GraphPad Prism 9.0.

## Results

### Method Development and Validation for Androgen Detection by β-RAM-HPLC

**Figure 2.**
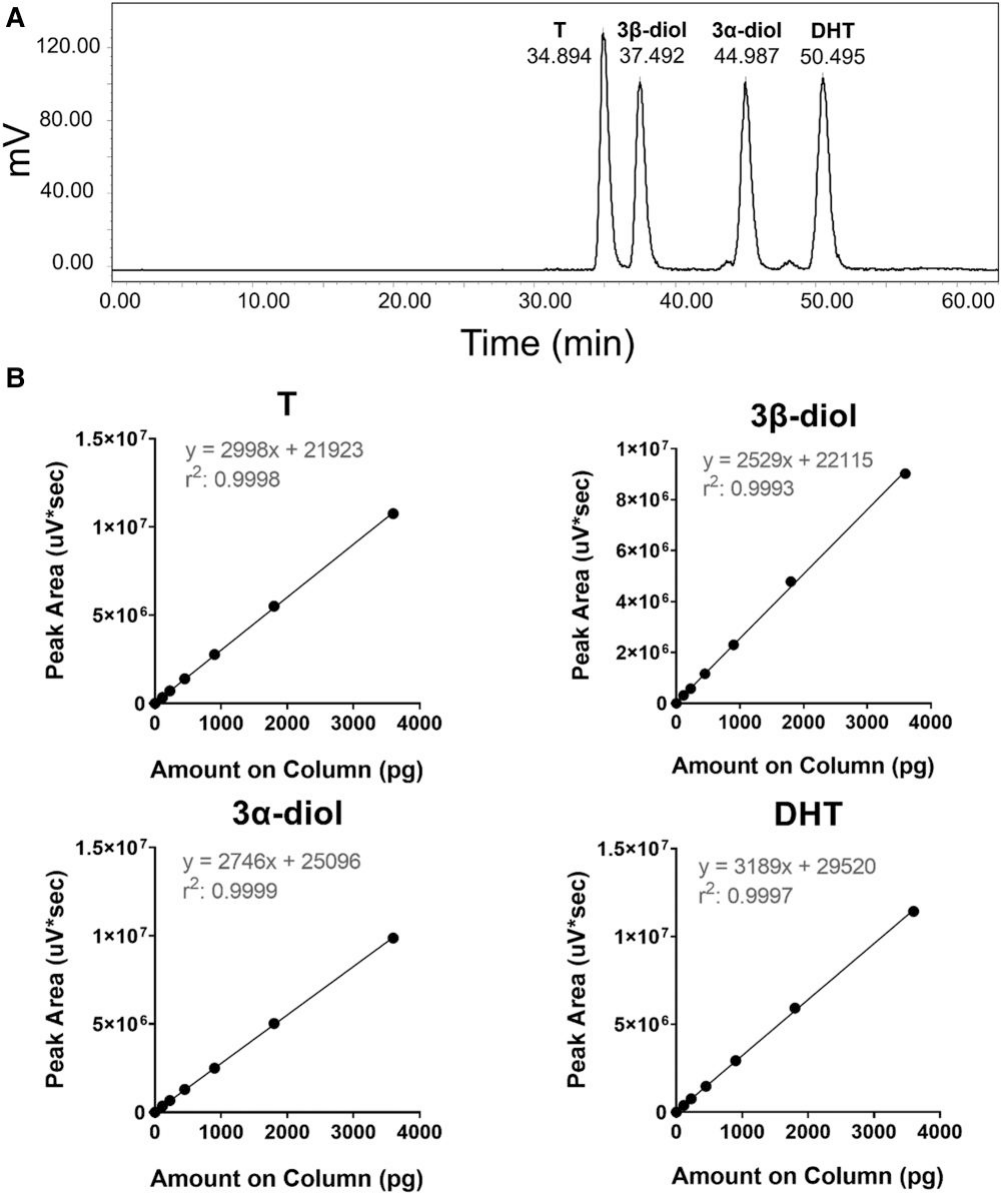
A, Radiochromatrographic separation of testosterone (T), 5α-DHT, 3α-diol, and 3β-diol on a C8 column. B, Calibration curves of T, 3β-diol, 3α-diol, and 5α-DHT.

Four closely related androgens (T, 5α-DHT, 3α-diol, and 3β-diol) were separated by a C8 column and detected by β-radioactive monitoring (Fig. 2A). Calibration curves of T, 5α-DHT, 3α-diol, and 3β-androstanediol standards were constructed in 60% ACN using 6 different amounts (112.5 pg, 225 pg, 450 pg, 900 pg, 1800 pg, 3600 pg) and a blank sample (0 pg). The calibration curves were linear based on their slopes and gave high correlation coefficients (*r*^2^) of 0.9993 to 0.9999 (Fig. 2B).

The lower limit of detection (LLOD) was defined as the lowest amount of targeted androgen that can be distinguished from its absence at a confidence of 95%. The lower limit of quantification (LLOQ) was defined as the lowest amount of targeted androgen that was experimentally quantified with an accuracy within 20% of theoretical value and a precision less than 20% of the coefficient of variation (CV). The determined values are listed in Table 1. The LLOQ was based on the ability to detect 15 to 20 pg (depending on analyte) with acceptable precision and accuracy based on a standard curve with the limits set as shown.

**Table 1.** Lower limit of detection, lower limit of quantification, accuracy, and precision determined from testosterone, 3β-diol, 3α-diol, and 5α-DHT

	T	3β-diol	3α-diol	5α-DHT
**LLOD, pg**	5.3	5.7	4.4	3.4
**LLOQ, pg**	20	20	20	15
**Accuracy,** **error %**	High: 4.9%	High: 0.5%	High: 3.2%	High: 2.6%
	Med: 0.6%	Med: 0.8%	Med: 0.1%	Med: 0.4%
	Low: 9%	Low: 11.8%	Low: 7.6%	Low: 10.2%
**Precision,** **% CV**	High: 2.4%	High: 2.3%	High: 1.7%	High: 0.8%
	Med: 0.2%	Med: 2.0%	Med: 3.6%	Med: 3.6%
	Low: 7.3%	Low: 7.7%	Low: 7.2%	Low: 6.1%

Abbreviations: CV, coefficient of variation; 5α-DHT, 5α-dihydrotestosterone; LLOD, lower limit of detection; LLOQ, lower limit of quantification; T, testosterone.

Quality control samples were prepared and analyzed on 3 nonconsecutive days to assess the precision and accuracy of targeted analytes. Precision and accuracy in 60% ACN were analyzed at 3 different quality control amounts (low: LLOQ 20 pg; medium: 300 pg and high: 1200 pg) each day. The accuracy was within 15% of the theoretical amount and the precision was less than 15% of CV, in compliance with US Food and Drug Administration Criteria for Bioanalytical Method Validation.

Reactions were replicated using unlabeled steroids, derivatized with picolinic acid, and subjected to stable isotope dilution LC-MS/MS using ^13^C-internal standards prepared in an identical fashion to the radioactive steroids. This approach provided rigorous analyte identity for the radiotracer method (Supplementary Fig. S3B [33]).

### Messenger RNA profile of Simpson-Golabi-Behmel Syndrome Adipocytes Through Differentiation Suggests Minimal 5α-Dihydrotestosterone Production

**Figure 3.**
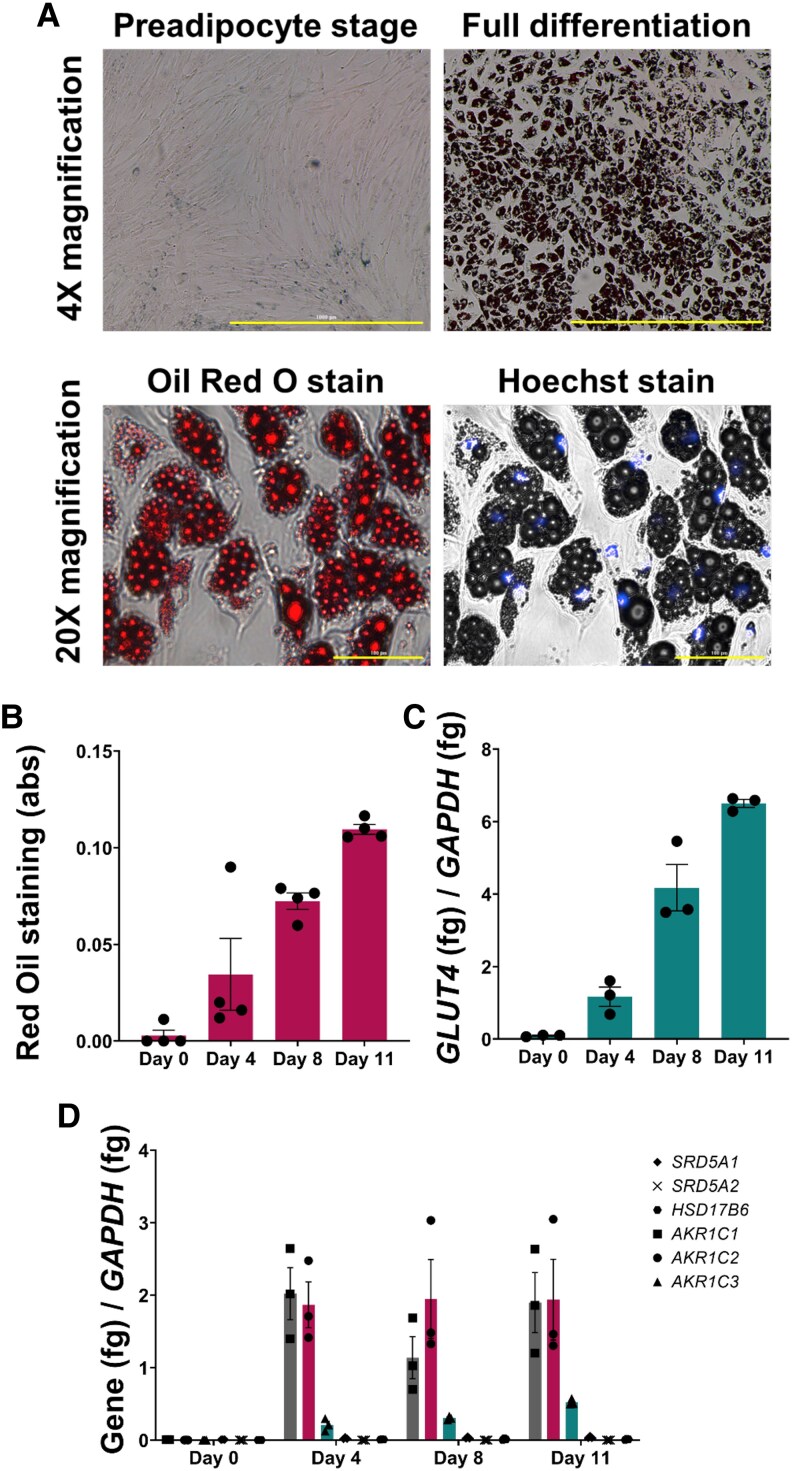
Simpson-Golabi-Behmel syndrome (SGBS) differentiation. A, Oil Red O staining of SGBS cells at preadipocyte stage and at full differentiated state at 4× magnification (1000 μm scale bar). At full differentiation and 20× magnification (100 μm scale bar), SGBS cells show lipid droplets and nuclei. B, Oil Red O staining extraction quantification through differentiation; n = 4. C. *GLUT4* messenger RNA (mRNA)/*GAPDH* mRNA quantification through differentiation; n = 3. D, *SRD5A1*, *SRD5A2*, *HSD17B6*, *AKR1C1*, *AKR1C2*, and *AKR1C3* mRNA quantification through differentiation, n = 3. All quantification is represented as mean ± SEM.

SGBS preadipocytes were differentiated to adipocytes by the Wabitsch protocol (Fig. 3A). Phenotypic (lipid accumulation) and genetic (*GLUT4* messenger RNA [mRNA]) markers showed a progressive linear increase over time during the differentiation period (Fig. 3B). mRNA levels of genes that regulate 5α-DHT levels were quantified. In general, transcripts of genes that increase 5α-DHT levels (*AKR1C3*, *SRD5A1*, *SRD5A2*, and *HSD17B6*) were significantly lower than transcripts of genes that decrease 5α-DHT levels (*AKR1C1* and *AKR1C2*) (Fig. 3C). Importantly, *AKR1C1*, *AKR1C2*, and *AKR1C3* transcripts were robustly induced during differentiation and the induction of *AKR1C1* and *AKR1C2* transcripts was higher than that observed for the *AKR1C3* transcript. Furthermore, while immunoblot detection was possible for AKR1C1/1C2 and AKR1C3 (Supplementary Fig. S4 [34]), this was not the case for other enzymes since there was significantly less mRNA as shown by reverse-transcription quantitative PCR. Additionally, publicly available proteomic data sets suggested low protein expression for these enzymes in adipocytes [35, 36].

### Minimal 5α-Dihydrotestosterone (5α-DHT) Synthesis Occurs in Simpson-Golabi-Behmel Syndrome Adipocytes While 5α-DHT Metabolism Occurs at Higher Rates

**Figure 4.**
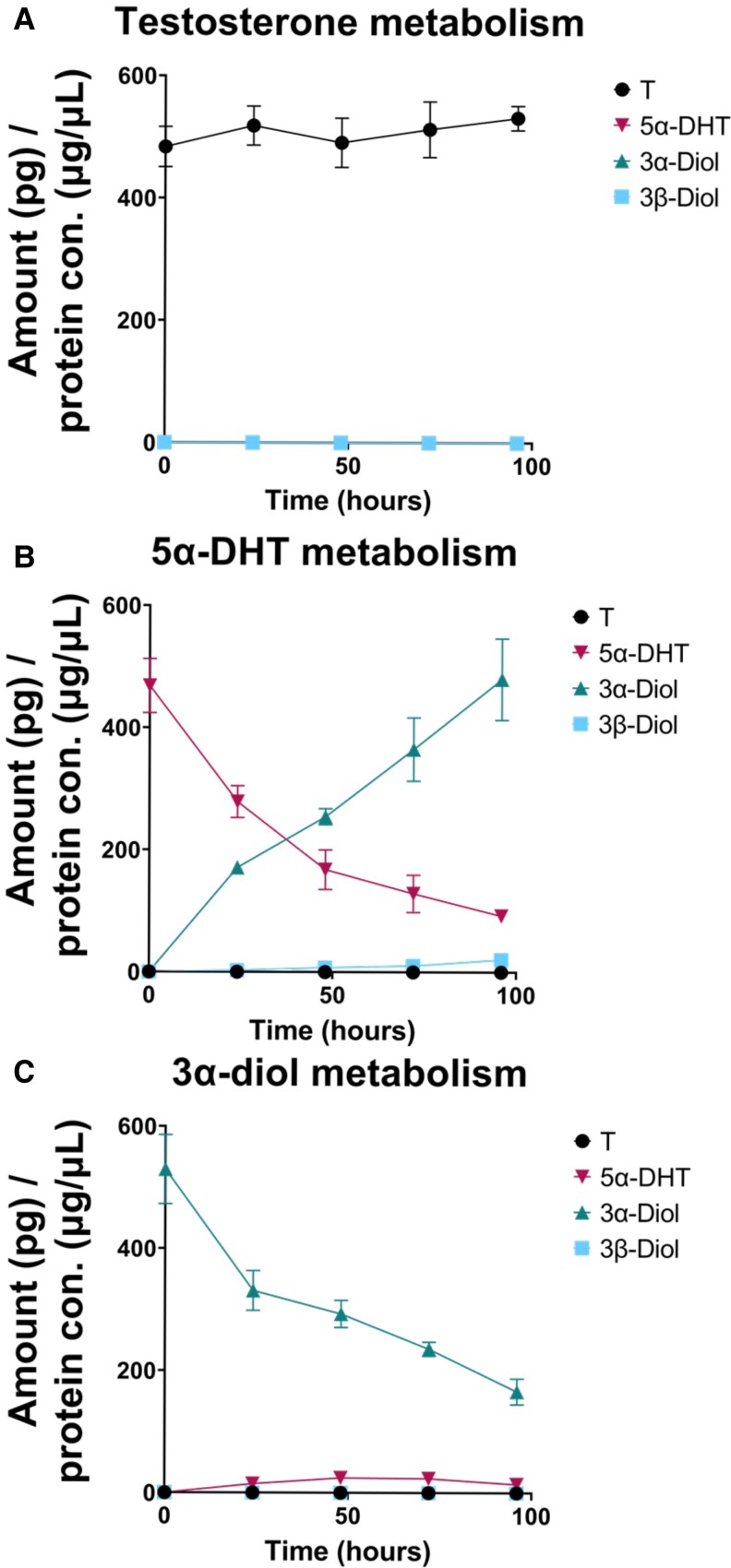
Metabolism of A, testosterone (T); B, 5α-DHT; and C, 3α-diol in a 96-hour time course. Protein concentration refers to cell lysates. All quantification is represented as mean ± SEM; n = 4.

Radiotracer experiments in SGBS adipocytes confirmed the genetic and proteomic data. When supplied with 10 nM T to measure its conversion to 5α-DHT, minimal T conversion occurred across 96 hours (Fig. 4A). These experiments contained 2.8 ng/mL of T, and the amount on column would be 840 pg with a LLOQ of 15 pg; the assay had the sensitivity to detect 1.7% conversion to DHT, yet none was detected. When supplied with 10 nM 5α-DHT to measure its conversion to 3α-diol and 3β-diol, 3α-diol was observed as the major reaction product, with low amounts of 3β-diol formation (Fig. 4B). When supplied with 10 nM 3α-diol to measure the back conversion of 3α-diol to 5α-DHT, minimal 5α-DHT formation was observed, while clearance of 3α-diol occurred (Fig. 4C).

### Messenger RNA Profile and Androgen Metabolism in Primary Adipocytes Corroborate Minimal 5α-Dihydrotestosterone (5α-DHT) Synthesis and Heightened 5α-DHT Metabolism

**Figure 5.**
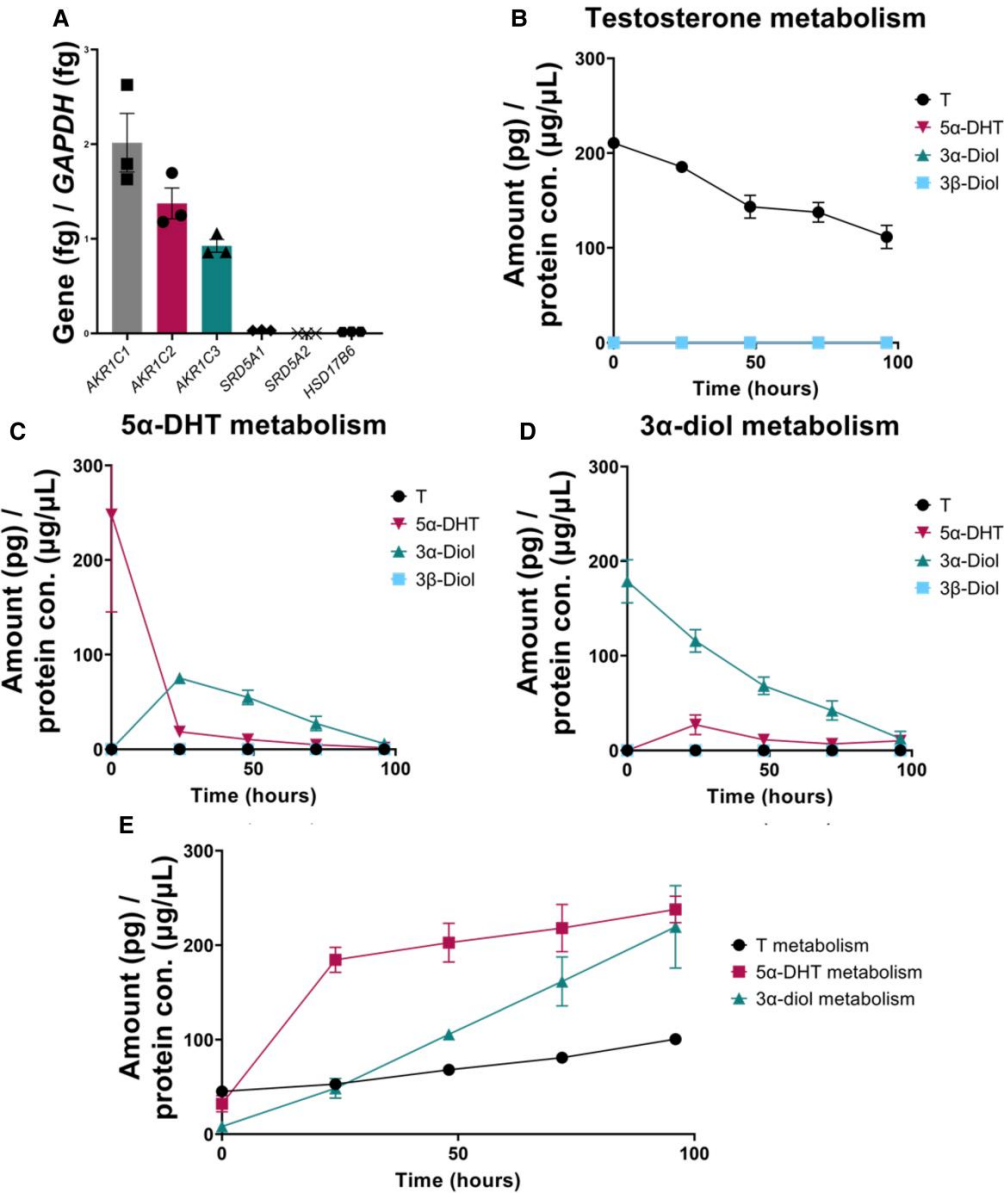
Fully differentiated primary cells. A, *SRD5A1*, *SRD5A2*, *HSD17B6*, *AKR1C1*, *AKR1C2*, and *AKR1C3* messenger RNA quantification at full differentiation. Metabolism of B, testosterone (T); C, 5α-DHT; and D, 3α-diol in a 96-hour time course. E, Radioactivity is present in aqueous layer of samples, an indicator of androgen elimination through conjugation. Protein concentration refers to cell lysates. All quantification is represented as mean ± SEM; n = 3.

To determine whether primary preadipocytes had the same pattern of androgen metabolism as the SGBS cells, preadipocytes were differentiated to adipocytes. At full differentiation, primary adipocytes have similar morphology to the SGBS adipocytes (Supplementary Fig. S5 [37]). mRNA of genes that regulate 5α-DHT levels showed that genes that metabolize 5α-DHT were expressed at higher levels than genes that biosynthesize 5α-DHT (Fig. 5A). mRNA transcript levels were comparable to the results seen in SGBS adipocytes (Table 2). Of those measured, *AKR1C3* mRNA was the only transcript that showed a statistically significant difference between SGBS cells and primary adipocytes at 0.53 ± 0.03 and 0.93 ± 0.07, fg/fg *GAPDH* (*P* ≤ .005), respectively.

**Table 2.** Values of messenger RNA ratio (fg/fg) of *SRD5A1*, *SRD5A2*, *HSD17B6*, *AKR1C1*, *AKR1C2*, and *AKR1C3* both in differentiated Simpson-Golabi-Behmel syndrome adipocytes and differentiated primary adipocytes

Gene	Reaction	Differentiated SGBS mRNA ratio, fg/fg *GAPDH^a^*	Differentiated primary mRNA ratio, fg/fg *GAPDH^a^*	Significant different, *P* ≤ .05
*AKR1C1*	5α-DHT → 3β-diol	2.25 ± 0.39	2.02 ± 0.30	No
*AKR1C2*	5α-DHT → 3α-diol	1.94 ± 0.56	1.38 ± 0.16	No
*AKR1C3*	4AD → 5α-DHT	0.53 ± 0.03	0.93 ± 0.07	Yes, *P* = .0049
*SRD5A1*	T → 5α-DHT	0.036 ± 0.008	0.034 ± 0.002	No
*SRD5A2*	T → 5α-DHT	Not detected	Not detected	No
*HSD17B6*	3α-diol → 5α-DHT	0.01 ± 0.005	0.02 ± 0.001	No

Abbreviations: mRNA, messenger RNA; SGBS, Simpson-Golabi-Behmel syndrome.

^a^Represented as mean ± SEM.

Radiotracer experiments in primary adipocytes gave findings consistent with those seen in SGBS cells in that 5α-DHT metabolism was favored over its synthesis. The media was sampled and extracted at 24-hour intervals over 96 hours, to quantify androgen levels. When supplied with 10 nM T, its conversion to 5α-DHT was minimal (Fig. 5B). The disappearance of T observed cannot be explained by its back oxidation to 4AD since this would have been detected in our radiochromatograms, in which there is a good separation of 4AD from the T peak. When supplied with 10 nM 5α-DHT to measure its conversion to 3α-diol and 3β-diol, 3α-diol was observed as the major reaction product, with no 3β-diol formation (Fig. 5C). When supplied with 10 nM 3α-diol to measure the back conversion of 3α-diol to 5α-DHT, minimal 5α-DHT was observed, while clearance of 3α-diol was robust (Fig. 5D). The decrease in overall radioactivity in the media was attributed to elimination of androgens through the formation of aqueous soluble radioactive metabolites (Fig. 5E). We subjected the aqueous phase to extraction plus and minus β-glucuronidase and aryl sulfatase treatment but were unable to recover additional radioactivity in the organic phase. Thus, while the accumulation of radioactivity in the polar fraction suggests phase 2 conjugation, this was not supported by the deconjugation reactions performed. We thus felt it was unnecessary to screen for specific uridine glucuronyl transferases or sulfotransferases within the cells. We have not determined whether radioactivity accumulates in the cells or lipid droplets since recovery of the radioactivity was greater than 80%.

## Discussion

We have characterized 5α-DHT synthesis and metabolism in a commonly used model of subcutaneous adipocytes, SGBS cells, and primary adipocytes collected from a lipoaspirate when they are fully differentiated. To accomplish this goal, we developed a method to quantify 5α-DHT, T, 3α-diol, and 3β-diol using radiotracers that allows for minimal sample manipulation and falls within the criteria for the US Food and Drug Administration Bioanalytical Method Validation. Our assay had an accuracy within 15% of the theoretical amount and had less than 15% CV. Our LLOD and LLOQ are between 4 and 5 pg and 15 and 20 pg, respectively, depending on the analyte and are an order of magnitude higher than SID-LC-MS/MS previously published by us [23]. But our method does not require derivatization or access to expensive mass spectrometry for routine metabolism studies, which may not be readily available. The sensitivity of the assay can be enhanced by reducing the amount of unlabeled steroid used as carrier and by using [^3^H]-T, which has a higher specific radioactivity than [^14^C]-T. The latter was used in this work based on its availability.

Radiochromatography in which HPLC coupled with β-RAM detection has been reported for measuring the backdoor pathway to 5α-DHT starting with [^3^H]-progesterone in the Tammar Wallaby [24] and in mouse testis [25], and in prostate cancer cell lines using [^3^H]-4-Ad to measure the 5α-Adione pathway to 5α-DHT [16]. However, in these reports the precision and accuracy of the method were not determined, and analyte identity was confirmed by comigration with authentic standards by thin-layer chromatography. Our method provides precision and accuracy and verified analyte identity by MS (see Supplementary Fig. S3 [33]).The presence of endogenous steroids is not accounted for in our assay.

Our expression data and metabolic tracing confirm that 5α-DHT is inactivated in subcutaneous adipocytes but it is not synthesized [38]. We found almost nondetectable levels of *SRD5A1* and *SRD5A2* transcripts in the two adipocyte cell lines. A proteomic search also found no SRD5A1, SRD5A2, or HSD17B6 expression in SGBS cells [35, 36]. These data support the lack of SRD5A1 expression in SAT from PCOS women [13, 39] and rule out different pathways to 5α-DHT formation. The inability to measure T conversion to 5α-DHT rules out the canonical pathway. The inability to detect *SRD5A1* and *SRD5A2* transcripts rules out the 5α-Adione pathway, which requires 5α-Adione. The lack of expression of *HSD17B6* and limited back conversion via 3α-diol shows that the backdoor pathway is not tenable.

Androgen inactivation is thus the predominant reaction in fat, particularly in abdominal SAT [39-41], as we have observed. SAT differentiation increases androgen inactivation, as seen before by the Blouin group [41, 42]. Negative associations between blood serum levels of 5α-DHT and total adipocytes have been noted in women with abdominal obesity or in premenopausal women [43-45], suggesting a protective [46, 47] role of SAT to inactivate 5α-DHT.

This raises the issue as to why DHEA was converted to 5α-DHT in microdialysis experiments using intra-SAT and why the ratio of 5α-DHT:T was higher in PCOS SAT [13]. SAT contains cell types other than adipocytes that could contribute to the formation of 5α-DHT observed, for example, fibroblasts, endothelial cells, pericytes, and immune cells known as the stromal vascular fraction. This maybe reminiscent of aromatase expression in human breast adipose stromal cells, which was identified as a source of estrogens in the breast as opposed to adipocytes [48].

T and 11-Keto-T can be formed in fully differentiated SGBS cells; however, 5α-DHT or 11K-5α-DHT were not detected [14]. We found that not only is 5α-DHT not formed but exogenously applied 5α-DHT is rapidly metabolized to 3α-diol and 3β-diol, implicating AKR1C2 and AKR1C1, respectively.

We have previously shown that *AKR1C3* is induced in SGBS cells by insulin. We now find that *AKR1C1* and *AKR1C2* are induced to greater extent than *AKR1C3* in both cell lines. *AKR1C3* is induced in part by nuclear factor-erythroid 2–related factor 2 (NRF2) [49]; *AKR1C1* and *AKR1C2* are also induced by NRF2 and has been corroborated in other cells by NRF2 knockout [50].

Transcript levels of *AKR1C1* and *AKR1C2* were comparable; however, 3α-diol was produced at a significantly higher amount than 3β-diol. This highlights the importance of metabolite quantification rather than relying on transcript levels alone to predict pathways of metabolism. Our results imply 5α-DHT inactivation via AKR1C2 as the major inactivation pathway in SAT.

We report the same patterns of 5α-DHT metabolism in two commonly used models of preadipocytes SGBS cells, and primary cells. The ancestry of both cell lines were different: SGBS were derived from a male infant patient of European descent, whereas the primary cells were isolated from a female adolescent patient of Hispanic descent and supports the rigor of our findings.

We acknowledge the limitations of the present work. All experiments were conducted on cell lines with exogenously supplied androgens. While androgen levels were within physiological relevant concentrations, future work detecting endogenous androgens in AT of PCOS patients would be of interest. We acknowledge that our radiolabeled steroids could be diluted by the endogenous unlabeled pool; however, this would not explain our results. We found that 5α-DHT is rapidly metabolized to 3α-diol, and there is an excellent product-precursor relationship in SGBS cells that would not be the case if there were considerable dilution by either endogenous 5α-DHT or 3α-diol. Dilution by endogenous 5α-DHT could account for the lack of ability to detect T conversion to 5α-DHT, but this is not occurring. Another limitation of the work is the fate of T, which in primary cells showed metabolism. The conversion of T to 4-Ad is not occurring since we would have detected [14C]-4-Ad in our radiochromatograms. The conversion of T to estradiol by aromatase cannot be ruled out. Others have measured the conversion of [1β-^3^ H]-4-Ad to estrone by following ^3^H_2_O release in SGBS cells [51]. We have also observed T radioactivity in the aqueous layer of samples supporting excretion of T through phase 2 conjugation reactions.

We conclude that subcutaneous adipocytes are a site of 5α-DHT inactivation through AKR1C2, and that inhibitors of AKR1C2 could contribute to hyperandrogenism and elevate 5α-DHT levels in an intracrine-dependent manner. We have found that perfluorooctanoic acid is a tight binding inhibitor of AKR1C2 and leads to increased 5α-DHT formation, suggesting that this endocrine-disrupting chemical could play a role in hyperandrogenism [52].

## Data Availability

Original data generated and analyzed during this study are included in this published article or in the data repositories listed in “References.”

## Abbreviations

3α-diol: 3α-androstanediol
4AD: 4-androstene-3,17-dione
Δ^5^-Adiol: 5-androstenediol
5α-Adione: 5α-androstane-3,17-dione
5α-DHT: 5α-dihydrotestosterone
AKR1C3: aldo-keto reductase family 1 member C3
AR: androgen receptor
AT: adipose tissue
cDNA: complementary DNA
CV: coefficient of variation
DHEA: dehydroepiandrosterone
DMEM: Dulbecco’s modified Eagle’s medium
FBS: fetal bovine serum
*GAPDH*: glyceraldehyde-3-phosphate dehydrogenase
HPLC: high-performance liquid chromatography
LLOD: lower limit of detection
LLOQ: lower limit of quantification
mRNA: messenger RNA
NRF2: nuclear factor-erythroid 2–related factor 2
PBS: phosphate-buffered saline
PCOS: polycystic ovary syndrome
PCR: polymerase chain reaction
PFA: paraformaldehyde
SAT: subcutaneous adipose tissue
SGBS: Simpson-Golabi-Behmel syndrome
SID: stable-isotope dilution
T: testosterone
VAT: visceral adipose tissue
